# INPART - a psycho-oncological intervention for partners of patients with haemato-oncological disease – study protocol

**DOI:** 10.1186/s12885-019-6094-2

**Published:** 2019-09-05

**Authors:** Inga Lorenz, Daniela Bodschwinna, Nina Hallensleben, Hartmut Döhner, Dietger Niederwieser, Tanja Zimmermann, Anja Mehnert, Harald Gündel, Jochen Ernst, Klaus Hoenig

**Affiliations:** 1 Department of Psychosomatic Medicine and Psychotherapy, University Medical Center Ulm, Albert-Einstein-Allee 23, 89081 Ulm, Germany; 2 Comprehensive Cancer Center Ulm, Ulm, Germany; 3 Department of Medical Psychology and Medical Sociology, University Medical Center Leipzig, Leipzig, Germany; 4 Department of Internal Medicine III, University Medical Center Ulm, Ulm, Germany; 5 Department of Haematology and Oncology, University Medical Center Leipzig, Leipzig, Germany; 6 Department of Psychosomatic Medicine and Psychotherapy, Hannover University Medical School, Hannover, Germany

**Keywords:** Psycho-oncology, Cancer, Group-intervention, Partner, Spouse, Depression, Anxiety, Quality of life, Dyadic coping

## Abstract

**Background:**

Suffering from cancer confronts both the patient and their partner with a number of psychosocial challenges in various aspects of their life. These challenges may differentially impact on quality of life, coping ability and compliance to treatment. This especially holds true for haemato-oncological diseases. To date, psychological interventions have predominantly been developed for oncological patients however specific interventions for partners of haemato-oncological patients are rare. In this study we aim to conduct a psycho-oncological group-intervention for partners of patients with haemato-oncological diseases. The aim of the intervention is to significantly reduce symptoms of depression and anxiety in the partners and the patient, as well as enhancing dyadic coping.

**Methods:**

The design of the INPART-study is an unblinded, randomised controlled trial with 2 treatment conditions (experimental and control) and assessments at baseline, 3 and 6 months. It will be conducted at three study centres: the university medical centre’s in Leipzig, Hannover and Ulm. The outcome criteria will be a reduction in depressive and anxiety symptoms as well as an improvement of dyadic coping.

**Discussion:**

This trial shall provide information regarding the efficiency of a psycho-oncological intervention for partners of patients with haemato-oncological diseases and give references to the possible outcome in terms of dyadic coping and the reduction of mental strain.

The study was supported by a grant from the German José Carreras Leukaemia Foundation.

**Trial registration:**

ISRCTN16085028; 20/03/2019.

## Background

Cancer does not only confront the patient with psychosocial challenges but also their partner [1, 2]. Pitceathly and Maguire [3] found that 20–30% of partners of cancer patients suffer from psychological stress rising to 50% when the cancer is highly progressive. This is particularly true to partners of haemato-oncological patients. Compared to other cancer types, the percentage of partners and caregivers of patients with haemato-oncological cancer affected with depression and anxiety is considerably higher [4]. Adjustment and anxiety disorders, depression, as well as sleeping disorders are among the most frequently reported distress-related symptoms. In addition to their concern about the patient, the uncertainty and the anxiety that arises from the diagnosis, they have to cope with changes which affect almost every aspect of their daily lives: their social role, financial situation, limitation of recreational activities and communication problems within their relationship [5]. Studies show that the psychological load for partners is comparable to that of the patients [6] and in some cases even higher [7, 8]. Simultaneously, the social environment does not perceive these problems adequately since the specific burden often remains invisible. This might be due to the fact that the main focus is on the patient. Partners tend to report their distress only rarely. As a result partners receive less social, health-care related and psychological support than the patient [9, 10] with negative consequences for both the patients and their partners as well as for dyadic coping (DC).

For haemato-oncological diseases the mentioned psychosocial burdens are particularly pronounced. Patients, as well as their partners, have to deal with significant limitations regarding their functionality and quality of life. The new situation requires high levels of adaptation by both the patient and their partner [11]. Haemato-oncological diseases are characterised by treatment interventions of long duration, that can cause severe therapy associated risks, the consequences of which can affect the patients and their partners years after treatment, for example in the case of stem cell transplantation [12, 13]. During treatment, the course of disease is highly unpredictable; people have to cope with the uncertainty of the success of the treatment as well as the possibility of a sudden life-threatening crisis. It is easily understandable that this can lead to severe emotional distress. Partners are often torn between caring for the patient and their other life commitments such as employment, child care and managing the household. Additionally, immunocompromised patients are required to make lifestyle changes such as restricting their social contact which can impact significantly on the mood of the person concerned. It is clearly evident that all of these difficulties influence the quality of the relationship between patient and partner, often resulting in profound communication difficulties. So far no data concerning haemato-oncological patients are available, however Manne et al. [14] showed in a group of breast cancer patients that the support of the partner is extremely important for the coping ability of the patient. This indicates strong support for the partner positively helps the patient coping ability. Conversely, a lack of support for partners can lead to maladaptive coping of the patient given that high scores of psychological stress in the partner have shown to have a negative consequence on quality of life and distress to the patient [15]. Considering the psychological stressors, described above, it is very likely that this is true, or even more pronounced, in haemato-oncological diseases.

Various psycho-oncological interventions aiming at reducing psychological load or an improvement in symptom management have been developed and successfully evaluated. Most of them focus on patients or the couple as a dyad [16, 17]. For partners there has been far less research [18] despite available evidence suggesting a better outcome for solely partner focused interventions than family based treatment - even for the patient [19]. There is no correlation between the patient’s course of disease and the perceived stress of the partner indicating an asynchronous process. Given that the needs and burdens of patients and partners differ over time, future interventions need to address the specific problems adequately both with regard to content and methods. With our INPART program we want to take a big step to closing this gap. In comparison to the established interventions INPART is addressing the partners solely by giving them the possibility to talk openly about the specific burdens in the safety of a homogenous group constellation. Established programs aim to reduce the psychological load of the patient or the partner, but fail to look at the interactions within the dyad. With the randomized controlled trial (RCT), described in the following, we want to investigate this reciprocal influence anticipating that by helping the partners to cope will have a positive influence on the dyadic coping and thus also on the psychological well-being of the patient.

### Aims of INPART

The aim of the present study is to examine the effectiveness of the INPART (INtervention for PARTners) program in reducing psychological distress (measured via depression and anxiety) and improving DC in partners and patients with haemato-oncological cancer in comparison to care as usual (CAU). The planned proceeding shall contribute to a better specificity of treatment and thereby to sustainable effects and significant benefits within the patient-partner-dyad.

### Intervention

The INPART program was created specifically for this study. It is a mixed intervention consisting of psychoeducational, cognitive-behavioural and imaginative elements. The decision, on which contents were to be included in the program, were based on research on the supportive care needs questionnaire for informal caregivers of cancer patients and cancer survivors [20]. The structure of the session is the same for each week, as can be seen in Table 1. At the end of the group phase each participant will get the chance for one or two individual psychotherapy sessions in order to face specific problems. Planned topics are: the individual handling of depression and anxiety, stress management, conflict training and managing difficult situations within the dyad (sexuality, communication problems).

**Table 1:** Overview of the standardized structure of the sessions

Treatment components	Content
Short opening discussion about today’s feeling	How do I feel at the moment? Questions about last session, if necessary
Monitoring home practice	Group conversation about the assigned exercises
Basic information	Psycho-education about today’s topic (for example communication within relationships)
Discussion of session topic	Talk between the participants led by the therapist: What do I experience as helpful? How do I cope with severe problems? Motivation: Partners shall learn from the model of others, how to cope more functionally and to be helpful for others
Practical exercise (incl. Home assignment)	Exercises for in-depth practice of the session topic, e.g., communication role play, integration of positive activities into daily routine
Outline for the next session	Short outlook on the topic of the next session
Relaxation/imagination/mindfulness	Introduction to established supportive interventions such as progressive muscle relaxation. Aims: Improvement of relaxation skills, resource activation and mindfulness practice to better cope with negative experiences, thoughts and feelings
Evaluation	Participant evaluates each session at the end

The program comprises of 5 weekly sessions lasting 1.5 h and additional home practice assignments. Groups consist of 6 to 8 participants. A presentation via Microsoft Powerpoint supports the structure of the course. In each session the participants receive material supporting the actual topic, a folder containing further information and home practice instructions for the forthcoming week. Table 2 shows the content of the INPART program per session.

**Table 2:** Structure and content of the INPART sessions and home practice

Session	Content	Form of relaxation techniques
1	● Introduction to the course and goals of INPART	Progressive muscle relaxation (PMR)
	● Information about the specific burdens of partners of patients with cancer	
	● Identification and activation of resources	
2	● Identification of negative appraisals, interpretations and meanings ● Recognizing and down-scaling of excessive self-expectations to facilitate daily life	Guided imagery: encouragement of a benevolent companion
3	● Communication within relationship: Introduction to basic rules of successful communication, non-verbal communication, gender differences in communication	Autogenic training
4	● Dealing with emotions, (focus on anxiety)	Mindfulness-based stress reduction
	● Promotion of functional anxiety management	
	*Aim*: Reduction of dysfunctional coping and regain of control	
5	*Final session*: ● Coping in daily life	Guided Imagery: safe place
	● Outlook: taking next steps	
	● Reflection of the program	

In order to test the standard operating procedures and evaluate the feasibility of the INPART program a pilot study was carried out. Between October and December 2017 patients and their partners were recruited as outlined for the INPART RCT. The participants were not screened for distress in order to be considered eligible for the study. All participants were assigned to the intervention condition as there was no control group. In January/February 2018 two trials were carried out in Ulm and Leipzig (*N* = 6). The pilot phase included a pre- and post- questionnaire and an evaluation of each INPART-session to find out which topics are relevant and to improve the final version of the intervention. Planned improvements were: less theory and more practical exercises, greater focus on self-care, a substantial reduction of the homework (experienced more as a burden by the participants) and the establishment of an additional session with a special topic (i.e. cancer and children). The pilot phase showed that the program was feasible and well received by the participants.

### Main study hypotheses

To alleviate depression as well as anxiety and to promote psychological well-being in partners of individuals with haemato-oncological disease the INPART-intervention was developed and will be tested in the proposed RCT. Principal hypothesis: A manualized group psychotherapy to treat clinically significant depressive and anxiety symptoms in partners of cancer patients with haemato-oncological disease will result in a greater reduction of depression and anxiety symptoms than in a control treatment of usual intervention (expected effect size ≥0,30) at three (t1) and six months (t2) after commencing intervention. In addition, the INPART program will lead to significant better DC. Secondary hypotheses: The INPART-intervention will result in better quality of life, better quality of the relationship and more self-efficacy than the control intervention. Between t1 and t2 a structured short-time psychotherapy in the intervention group will lead to further positive effects in the dependent variables compared to that of the treatment as usual group.

## Methods / design

### Study design

**Fig. 1:**
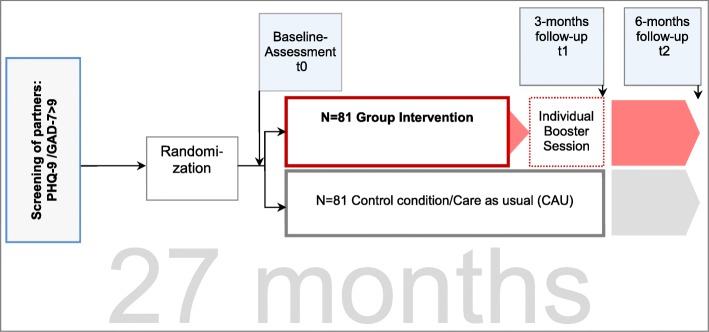
Study procedure

The design of the INPART-study will be an unblinded, randomized controlled trial with 2 treatment conditions (experimental and control) and assessments at baseline, 3 and 6 months. It will be conducted at three study centers: the university medical centre in Leipzig, Hannover and Ulm. Following an initial screening procedure, eligible partners (necessary requirement PHQ-9 or GAD-7 > 9) will be randomized into one of two groups at which baseline-assessment will take place (see Fig. 1). Participants will be either assigned to the intervention group (IG) or to the control group (CG). T1 is 3 months after the start of the intervention. Directly after the intervention, before t1, subjects of the intervention group will have the possibility of one additional single session in order to talk about individual topics/problems (i.e. hope, death and loss). Subjects of the control group will be treated as usual (optional unspecific contacts with a psycho-oncologist). At all assessments partners and patients are asked to fill out the questionnaires. The study protocol has been approved by the ethical review boards of each of the three centres. The registered project number is DJCLS R 12/36.

### Participants and procedure

**Fig. 2:**
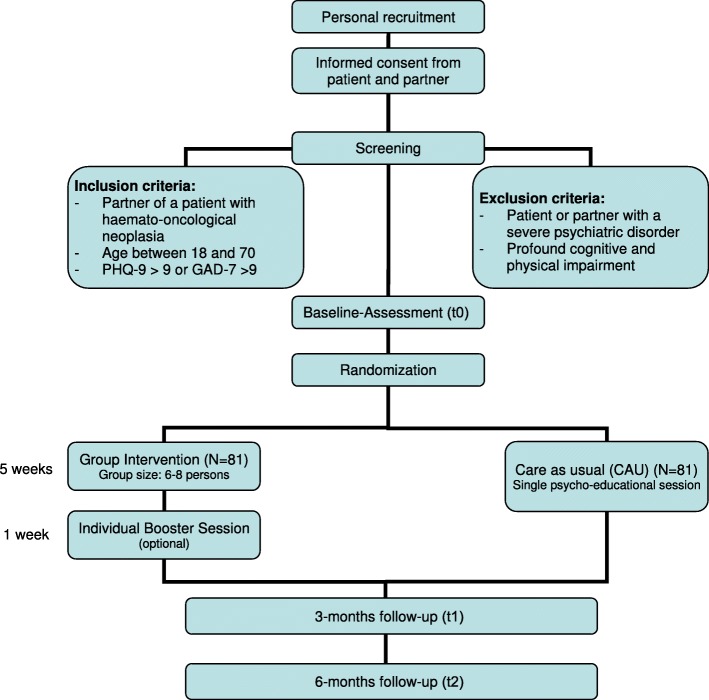
INPART flow diagram

Patients and their partners are recruited at the departments of haemato-oncology at the three university medical centres (Leipzig: University Cancer Center Leipzig (UCCL), Hannover: Oncology Center (OZ), Ulm: Comprehensive Cancer Center Ulm (CCCU)) by a student assistant at each centre. Inclusion criteria requires a haemato-oncological diagnosis, being in a relationship and written informed consent for participation in the study. Partners will be contacted via the patient. Once consent from the patient has been given, partners are asked for their informed consent. Partners are randomly assigned to one of the two study groups. For participant flow see Fig. 2. For an overview of measurements and corresponding time frames see Table 3.

**Table 3:** Measurements and corresponding time points for patient and partner

Instrument	Target	Screening		Baseline (t0)		3-month follow-up (t1)		6-month follow -up(t2)	
		Partner	Patient	Partner	Patient	Partner	Patient	Partner	Patient
Demographics		x	x						
Clinical characteristics			x		x		x		x
PHQ-9	depression	x	x	x	x	x	x	x	x
GAD-7	anxiety	x	x	x	x	x	x	x	x
BFI	Fatigue			x	x	x	x	x	x
SF-12	Quality of Life			x	x	x	x	x	x
IPC	self-efficacy			x	x	x	x	x	x
ECR-RD	Quality of Relationship			x	x	x	x	x	x
DCI	DC			x	x	x	x	x	x

Note. *PHQ-9* = Patient Health Questionnaire, *GAD-7* = Generalization Anxiety Disorder Scale, *BFI* = Brief-Fatigue-Inventory, *SF-12* = Health Survey 12, *IPC* = Internal, Powerful Others, and Chance Scale, *ECR-RD* = Experience in close Relationships-Revised, *DCI* = Dyadic Coping Inventory

### Eligibility for study participation

We include patients who are diagnosed with a confirmed haematological neoplasia: incidences up to one year after diagnose or relapse, ICD-10 diagnoses: C81-C96 and D46, which are: Hodgkin’s lymphoma, Non-Hodgkin’s lymphoma, multiple myeloma, myelodysplastic syndrome, acute or chronic leukaemia. We include patients who have a partner. Patients and partners need to be between 18 and 70 years. In order to be eligible, partners must be mentally and physically able to attend the program. Treatment modality and phase of illness is negligible for participation. Partners will be assigned to one of two study groups, given that PHQ-9 > 9 (depression) and / or GAD-7 > 9 (anxiety). Exclusion criteria for patients and partners are: a) severe psychiatric disorders, b) profound cognitive and physical impairment. An age limitation of 70 years aimed to minimise possible age-related comorbidities or mobility limitations. Exclusion and inclusion criteria will be checked in the patients’ records or in discussion with the attending physician.

### Randomization procedure

Study entry requires a positive screening result and consent from the eligible partners. During screening partners are asked to fill out the PHQ-9 (depression) and the GAD-7 (anxiety). The necessary criteria for randomization is a cut-off score > 9 in at least one of the two scales (see [21, 22]). Randomization will be carried out on a basis of fixed blocks of flexible length. Within one block the assignment is balanced meaning that the number of interventions and control subjects are the same. The different length of the blocks is chosen to further reduce predictability of the assignment.

Possible selection effects will be documented to analyse their effect on the generalization of the results. Drop-out participants will be included in an intent-to-treat-analysis. To control for potential undesired side effects, other psycho-oncological or complementary interventions are recorded and considered in the analysis as well as information from the treatment process. In the case of events during the course of the study which may influence the therapeutic implications of the intervention negatively i.e. newly occurring exclusion criteria for example cognitive impairments or mobility deterioration, participants will be excluded. Psycho-oncological or psychiatric help is ensured by the local psychosomatic consultation-liaison and the psychiatric consultation services.

### Assessment

#### Baseline assessment

Following initial screening data collection will take place at three different intervals: baseline (t0), 3-months follow-up (t1) and 6-months follow-up (t2). in both groups. In addition to demographic and clinical characteristics, information about the quality of the relationship, dimension of the psychological burden (fatigue, anxiety, intensity of depression), quality of life, need for support and DC skills are collected. The applied questionnaires are well established. They have been standardized and validated. Table 3 shows the assessment tools and time frames at which the questionnaires are to be presented to patients and partners.

#### Follow-up assessments

Follow-up assessments will take place post intervention around three months after start of the intervention and at a six-month follow-up appointment. The participants will receive paper versions of the questionnaires along with return envelopes. In the case of drop-out, the researcher tries to contact the participant by phone to complete a minimum set of outcome measures and to ascertain the reasons for the drop-out.

#### Control group

The control group receives one structured psycho-oncological consultation (Control condition/Care as usual), which is regularly conducted by trained psycho-oncologists The duration of the consultation is approximately 30 min. Example issues discussed consist of the role of partner within the course of the illness including their own specific burdens and possibility of support. In addition they will receive a freely accessible booklet from the German Cancer Aid associated to their specific oncological disease.

#### Measures

Table 3 shows all study measures that are planned in the RCT. Demographic information such as age, sex, marital status, education, occupational situation in addition to treatment and disease related variables i. e. cancer diagnosis, date of diagnosis, past and current medical treatments, are collected using a standardized questionnaire.

The Depression module of the *Patient Health Questionnaire (PHQ-9)* is a valid self-report screening tool for depression. It consists of nine items each representing one criterion for major depression according to the Diagnostic and Statistical Manual of Mental Disorders, 4th Revision (DSM-IV). Items are scored on a four-point Likert scale from 0 (“not at all”) to 3 (“nearly every day”) with a total range from 0 to 27. Higher values indicate increased severity of symptoms. Scores > 9 indicate the presence of depression and further diagnostic assessment is recommended. Reliability and validity of the PHQ-9 is very good, and is preferably used compared to that of other screening instruments when evaluated with diagnostic criteria provided by the DSM-IV as a reference standard [23–25].

The *Generalized-Anxiety-Disorder-Questionnaire (GAD-7)* is a module of the Personal Health Questionnaire (PHQ) assessing generalized anxiety disorder. It consists of 7 items each of which reflect one DSM-IV criteria for generalized anxiety disorder. Items are scored on a four-point Likert scale from 0 (“not at all”) to 3 (“nearly every day”), resulting in a total range from 0 to 21. Scores from 0 to 4 denote an absence of anxiety while scores from 5 to 9, 10–15, and a score higher than 15 correspond to mild, moderate and severe levels of anxiety respectively. A cut-off value of ≥10 has been recommended to screen for any anxiety disorders. There is a high internal consistency of the German version of the questionnaire with Cronbach’s α =0.89. The German version has been validated and is commonly used in various diseases [22, 26].

The *Brief-Fatigue-Inventory (BFI)* is a short validated tool assessing the severity of fatigue in cancer patients and their partners. In the first step participants state whether they felt unusually tired or fatigued in the past week. In the second step 9 questions measure the experienced fatigue and its influence on aspects of the participants’ daily lives in the last 24 h. The BFI is rated on an eleven-point Likert scale ranging from 0 (“no fatigue”, “does not interfere”) to 10 (“as bad as you can imagine”/“completely interferes”). The mean BFI is calculated over the 9 questions with 1–4 indicating mild, 5–6 moderate, and 7–10 severe fatigue. The German version of the questionnaire shows a high internal consistency with Cronbach’s α = 0.92 [27].

The *Experience in Close Relationships scale (ECR-RD)* is a self-report questionnaire. It assesses patient and partner experiences in close romantic and non-romantic relationships and consists of two subscales: anxiety and avoidance. Items are scored on a seven-point Likert-scale ranging from 1 (“disagree”) to 7 (“agree”). Higher scores on one or both subscales indicate high attachment insecurity. Both subscales show high internal consistency with Cronbach’s α = 0.91 (anxiety) and α = 0.88 (avoidance) [28, 29].

The *Internality, Powerful others, and Chances scale (IPC)* assesses self and environment related cognitions on three dimensions: internality (conviction in having control over one’s own life), social related externality (having the impression of being dependent on other more powerful persons) and fatalistic externality (conviction that life is mainly influenced by fate/coincidence). It consists of 24 items which are scored on a six-point Likert-scale from 0 (“very wrong”) to 5 (“very correct”) [30].

The *Dyadic Coping Inventory (DCI)* is a self-administered instrument with 37 items which assesses stress and coping abilities within relationships when one partner is stressed. The DCI comprises four different dimensions/subscales: supportive DC e.g. ‘I show empathy and understanding’, delegated DC e.g. ‘I take on things that my partner would normally do in order to help him/her out’, negative DC e.g. ‘I blame my partner for not coping well enough with stress’, and stress communication e.g. ‘I let my partner know that I appreciate his/her practical support, advice or help’ as well as common DC e.g. ‘We try to deal with the problem together and look for concrete solutions’. It is also possible to assess the overall evaluation of DC which contains the satisfaction with DC and the evaluation of its efficacy. Each partner rates his own DC as well as the perceived DC of his partner. The items are rated on a five-point Likert scale from 1 (“very rarely”) to 5 (“very often”). The German version of the questionnaire shows a high internal consistency with Cronbach’s α = 0.91 [31–33].

The *Health Survey 12* (SF-12) is a general health questionnaire that assess health-related quality of life (QoL). It gives information about the health status of a person on eight different dimensions (physical functioning, physical role functioning, bodily pain, general health, vitality, social functioning, emotional role functioning and mental health). The dimensions can be summarized on two scales: the “mental health composite scale” and the “physical health composite scale”. The questionnaire consists of 12 self-administered items, which are rated on a 3, 5 or 6-point Likert scales. The two composite scales range from 0 very low Quality of Life (QoL) to 100 very high QoL. Most of the subscales show a high internal consistency with Cronbach’s α > 0.70 [33, 34].

#### Individual evaluation of therapy sessions

To evaluate each of the INPART group sessions we developed a specific questionnaire for each of the sessions asking to what extent the presented topic reflects the current concern of the partner and, at the same time, to what extend they perceived the therapy sessions helpful. An extra question aimed at the specific exercises of relaxation, resource activation and mindfulness in the session e.g., “To what extent could you get involved in the progressive muscle relaxation?”. For each of the evaluation questionnaires extra space is provided for individual feedback. We also developed a specific evaluation questionnaire for the therapists asking to what extent they felt to be supportive for the partners in respect to the relevant aspects.

#### Quality standards and therapist training

##### The INPART treatment manual

The INPART treatment manual was specifically developed. Until now there is no comparable group intervention program for partners of haemato-oncological patients available.

##### Therapist qualifications, INPART-training, and supervision

The INPART- courses are taught by professional psychotherapists and psycho-oncologists with specific psycho-oncological experience with at least two years of professional experience and a special certificate in psychosocial oncology. They will receive special training for the INPART program and will be supervised after each of the five sessions. The therapists of the control group will not receive any additional training but will also receive regular experienced supervision.

### Statistical methods

#### Power calculation

The sample size is determined based on a two-way ANOVA with two between-group factors (1: group, 2: centres) and a within-subject repeated measures factor (Time). Outcome criteria is the reduction of depression and anxiety (PHQ-9 and GAD-7). Alpha is defined with 0.05, Beta with 0.80. Effect size f (F-Test or ANOVA) is with 0.25 on an average level. Thus a sample size of *N* = 162 persons is necessary for finding a significant difference between intervention and control group (calculated with Gpower). The feasibility of the sample is based on the number of patients during 2016 in all three centres. According to the clinical cancer register there are 284 incidences per year in Leipzig, 292 in Hannover, and 372 in Ulm. Given an inquiry period of 27 months this would mean 639 patients in Leipzig, 657 in Hannover, and 837 in Ulm. Of these patients 25% will not fulfil the inclusion criteria (e.g. not living in a relationship, insufficient language skills) which means that 479 possible participants in Leipzig, 492 in Hannover, and 627 in Ulm can be addressed. After positive screening (50%) and acceptance of randomization (another 50%) there will be 119 participants in Leipzig, 123 in Hannover, and 156 in Ulm. The expected number of 4 intervention groups of 6 to 8 persons per centre can thus be easily achieved. The same number of groups / participants will be needed for the control group of the study.

#### Statistical analyses

In order to quantify the collected data descriptively summary statistics like mean values, variances, measures for value distribution and frequencies will be calculated for characteristics like frequencies of cancer types, gender, age and quality of relationship. For exploratory data analysis cross table evaluations will be performed including sociodemographic and disease specific control variables in order to find possible age or educational related effects. Correlation analyses will be used to assess the strength of associations between variables. At the interferential level mean comparisons via t-test or Mann-Whitney-U-Test will be employed to assess the efficacy of INPART in comparison to the control group at three different time intervals. We expect that that the level of depression and anxiety will change over time and a reduction is highest in the treatment group. Main analyses will be performed using a multivariate analysis of variance with repeated measures (MANOVA). In addition, multiple regression analysis will be applied to identify predicting factors (Table 4).

**Table 4:** Descriptive and inferential statistics

Descriptive statistics	˗ Exploratory data analysis (value distribution, analyses of variance)
	˗ Cross table evaluation incl. Control variables (sociodemographic, disease specific)
	˗ Correlation analysis
Inferential statistics	˗ Comparisons of means (t-test or Mann-Whitney-U-test)
	˗ Multivariate analyses of variance (for multiple dependent variables), general linear model
	˗ (Multiple) logistic Regression for identification of predictors and determination of effectiveness
	˗ Actor-partner-interdependence model in order to investigate reciprocal influence within the dyad

## Discussion

Partners of patients with a haemato-oncological disease are often confronted with diverse psychosocial challenges with distress scores being equal to or sometimes even above those of the patients. At the same time psychosocial or psycho-oncological interventions for this target group are rare. So far, most of the developed and evaluated interventions are aimed at patients or couples although there is some evidence pointing to an outcome advantage for pure partner focused interventions. The present study is aimed at empirically closing this gap.

Data from the previous bi-centric pilot study at the university medical centres of Ulm and Leipzig demonstrated the feasibility of the INPART-program and proved that the chosen topics are relevant to the partners (see above). Communication within the relationship and dealing with negative feelings were reported by the partners to be amongst the most relevant components. Many of the partners reported difficulties in talking about their emotions. Specifically fear and anxiety were reported to be particularly difficult to talk about due to feeling the need to protect their ill spouses. During the program they learnt that it can be beneficial to talk about their feelings leading to a decreased feeling of loneliness on both sides. In the presented RCT we plan to measure the level of perceived loneliness in the partners prior to and after the intervention. Hawkley and Cacioppo [35] describe in their review how perceived loneliness increases vigilance for threat and heightens feelings of vulnerability which can have negative effect on cognitive, behavioural and physiological functioning increasing morbidity and mortality. With this in mind it seems crucial to help patients and their partners to overcome such feelings.

Another highly valued component of the INPART-program was the unit comprising the practical exercises (progressive muscle relaxation (PMR), resource activation, mindfulness) where the participants were introduced to these diverse methods. Instead of just learning one alternative (e.g. PMR), a broad introduction was given so that the partners can choose at the end of the program which method suits them best in order to foster their motivation to incorporate into daily life. Feedback from the participants showed us that this was very well received.

Expectedly the feasibility study demonstrated that recruitment of participating partners was challenging (participants quote among 10%). There were several obstacles for participation in the program. Firstly, contacting the partners is dependent on prior contact with the patients. Informing the patient and leaving a leaflet may not be sufficient. Possible questions which occur: Do the patients pass on the information to their partners? If so, do the partners realise that this is something potentially beneficial to them? Given the rather large catchment areas of both centres in Leipzig and Ulm, many partners considered the distance from their hometown to the clinic as a critical barrier for participation. Understandably the willingness of partners to afford a one-hour drive one-way weekly was rather low – particularly when the patient was not in the clinic anymore. Distance to the centre would likewise be a critical factor with respect to the travel ability of participating patients. In addition to these more practical reasons we also experienced prejudices against psychological interventions both by partners and/or patients.

Keeping these experiences in mind effective recruitment will be crucial for the success of the presented study. We plan to strengthen the co-operation with the treating physicians, aiming at a standardised information talk in the first days of the hospital stay for all patients/partners who fulfil the inclusion criteria. In this information talk, we will focus attention on both the supportive role of partners and on their respective need for psychosocial support. It will be highlighted that participation in the InPart-program might not only be beneficial for partners, but indirectly also for the patients. Since the nursing staff is working closely with the patients they also play an important role in motivation and should therefore be actively involved in the recruitment process. Thereby, we hope to much better reach all potential participants. In order to increase the number of study participants within a shorter period of time, we decided to include an additional university cancer centre.

To date partners receive minimal social, health-care related and psychological support even though their psychological load is as high as that of the patients. With the presented study we want to counteract this observation by achieving decreased levels of depression and anxiety as well as increasing DC. Helping the partners to cope will have positive influence in the coping ability of the patient. Since INPART is planned as a group-intervention it also has valuable economic and synergistic advantages.

## Data Availability

Not applicable.

## Abbreviations

ANOVA: Analysis of variance
BFI: Brief-Fatigue-Inventory
CAU: Care as usual
DC: Dyadic coping
DCI: Dyadic Coping Inventory
DSM-IV: Diagnostic and Statistical Manual of Mental Disorders, 4th Revision
ECR-RD: Experience in Close Relationships scale - revised
GAD-7: Generalized-Anxiety-Disorder-Questionnaire
IPC: Internality, Powerful others, and Chances scale
MANOVA: Multivariate analysis of variance
PHQ-9: depression module of the Patient Health Questionnaire
PMR: Progressive muscle relaxation
QoL: Quality of life
RCT: Randomized controlled trial
SF-12: Health Survey 12
T1: Time point 1 of data acquisition

## References

[1] Hagedoorn M, Sanderman R, Bolks HN, Tuinstra J, Coyne JC (2008). Distress in couples coping with Cancer: a meta-analysis and critical review of role and gender effects. Psychol Bull.

[2] Ernst J, Weißflog G. Family, relationship and cancer. In: Mehnert A, Koch U, editors. Handb Psycho-Oncol. Göttingen: Hogrefe; 2016:284–95.

[3] Pitceathly C, Maguire P (2003). The psychological impact of cancer on patients’ partners and other key relatives:a review. Eur J Cancer.

[4] Lambert SD, Girgis A, Lecathelinais C, Stacey F (2013). Walking a mile in their shoes: anxiety and depression among partners and caregivers of cancer survivors at 6 and 12 months post-diagnosis. Support Care Cancer.

[5] Popek V, Hönig K (2015). Cancer and family: tasks and stress of relatives. Nervenarzt..

[6] Mitchell AJ, Ferguson DW, Gill J, Paul J, Symonds P (2013). Depression and anxiety in long-term cancer survivors compared with spouses and healthy controls: a systematic review and meta-analysis. Lancet Oncol.

[7] Kayser K, Watson LE, Andrade JT (2007). Cancer as a “we-disease”: examining the process of coping from a relational perspective. Fam Syst Heal.

[8] Carlson Linda E., Bultz Barry D., Speca Michael, St. Pierre Mereille (2000). Partners of Cancer Patients. Journal of Psychosocial Oncology.

[9] Rosenberger C, Höcker A, Cartus M, Schulz-Kindermann F, Härter M, Mehnert A (2012). Relatives and patients in the outpatient psycho-oncological care: access paths, psychological burdens and support needs. PPmP-Psychotherapie· Psychosom Medizinische Psychol.

[10] Lambert SD, Harrison JD, Smith E, Bonevski B, Carey M, Lawsin C (2012). The unmet needs of partners and caregivers of adults diagnosed with cancer: a systematic review. BMJ Support Palliat Care.

[11] Bishop MM, Beaumont JL, Hahn EA, Cella D, Andrykowski MA, Brady MJ (2007). Late effects of cancer and hematopoietic stem-cell transplantation on spouses or partners compared with survivors and survivor-matched controls. J Clin Oncol.

[12] Seeber S, Schütte J. Therapy concepts oncology: Springer; 2013.

[13] Rodin G, Yuen D, Mischitelle A, Minden MD, Brandwein J, Schimmer A (2013). Traumatic stress in acute leukemia. Psycho-Oncology..

[14] Manne SL, Pape SJ, Taylor KL, Dougherty J (1999). Spouse support, coping, and mood among individuals with cancer. Ann Behav Med.

[15] Manne Sharon L., Ostroff Jamie, Winkel Gary, Grana Generosa, Fox Kevin (2005). Partner unsupportive responses, avoidant coping, and distress among women with early stage breast cancer: Patient and partner perspectives. Health Psychology.

[16] Northouse LL, Mood DW, Schafenacker A, Kalemkerian G, Zalupski M, LoRusso P (2013). Randomized clinical trial of a brief and extensive dyadic intervention for advanced cancer patients and their family caregivers. Psycho-Oncology..

[17] Sheinfeld Gorin S, Krebs P, Badr H, Janke EA, Jim HSL, Spring B (2012). Meta-analysis of psychosocial interventions to reduce pain in patients with cancer. J Clin Oncol.

[18] Wootten AC, Abbott JM, Farrell A, Austin DW, Klein B (2014). Psychosocial interventions to support partners of men with prostate cancer: a systematic and critical review of the literature. J Cancer Surviv.

[19] Hartmann M, Bäzner E, Wild B, Eisler I, Herzog W (2010). Effects of interventions involving the family in the treatment of adult patients with chronic physical diseases: a meta-analysis. Psychother Psychosom.

[20] Sklenarova H, Krümpelmann A, Haun MW, Friederich H, Huber J, Thomas M (2015). When do we need to care about the caregiver? Supportive care needs, anxiety, and depression among informal caregivers of patients with cancer and cancer survivors. Cancer..

[21] Manea L, Gilbody S, McMillan D (2012). Optimal cut-off score for diagnosing depression with the patient health questionnaire (PHQ-9): a meta-analysis. Can Med Assoc J.

[22] Löwe B, Decker O, Müller S, Brähler E, Schellberg D, Herzog W (2008). Validation and standardization of the generalized anxiety disorder screener (GAD-7) in the general population. Med Care.

[23] Spitzer RL, Kroenke K, Williams JBW (1999). Validation and utility of a self-report version of PRIME-MD: the PHQ primary care study. Jama..

[24] Löwe B, Spitzer RL, Zipfel S, Herzog WPRIMEMD. Patient health questionnaire (PHQ)-German version 2nd edn: Pfizer Karlsruhe; 2002.

[25] Löwe B, Kroenke K, Herzog W, Gräfe K (2004). Measuring depression outcome with a brief self-report instrument: sensitivity to change of the patient health questionnaire (PHQ-9). J Affect Disord.

[26] Spitzer RL, Kroenke K, Williams JBW, Löwe B (2006). A brief measure for assessing generalized anxiety disorder: the GAD-7. Arch Intern Med.

[27] Shahid A, Wilkinson K, Marcu S, Shapiro CM, Shahid A, Wilkinson K, Marcu S, Shapiro C (2011). Brief fatigue inventory. STOP, THAT one hundred other sleep scales.

[28] Brennan KA, Clark CL, Shaver PR, Simpson J, Rholes W (1998). Self-report measurement of adult attachment: an integrative overview. Attach theory close relationships.

[29] Lo C, Walsh A, Mikulincer M, Gagliese L, Zimmermann C, Rodin G (2009). Measuring attachment security in patients with advanced cancer: psychometric properties of a modified and brief experiences in close relationships scale. Psychooncology..

[30] Levenson H. Differentiating among internality, powerful others, and chance. In: Lefcourt HM, editor. Res with locus control Constr: Academic Press; 1981.

[31] Bodenmann G (2008). Dyadisches coping Inventar: DCI Testmanual.

[32] Ledermann Thomas, Bodenmann Guy, Gagliardi Simona, Charvoz Linda, Verardi Sabrina, Rossier Jérôme, Bertoni Anna, Iafrate Raffaella (2010). Psychometrics of the Dyadic Coping Inventory in Three Language Groups. Swiss Journal of Psychology.

[33] Ernst J, Hinz A, Niederwieser D, Döhner H, Hönig K, Vogelhuber M (2017). Dyadic coping of patients with hematologic malignancies and their partners and its relation to quality of life – a longitudinal study. Leuk Lymphoma.

[34] Ware JE, Kosinski M, Keller SD (1996). A 12-item short-form health survey: construction of scales and preliminary tests of reliability and validity. Med Care.

[35] Hawkley LC, Cacioppo JT (2010). Loneliness matters: a theoretical and empirical review of consequences and mechanisms. Ann Behav Med Oxford University Press.

